# Book Review

**DOI:** 10.1120/jacmp.v7i4.2383

**Published:** 2006-11-28

**Authors:** David L. Goff

**Affiliations:** ^1^ President Medical and Radiation Physics Inc. San Antonio Texas

## Abstract

***Medical Health Physics***, David Medich & Christopher Martel Editors, Medical Physics Publishing, ISBN 1‐30524‐31‐5, $75.00

*Medical Health Physics* is the textbook used for the 2006 Health Physics Society Summer School. The book is intended to provide a practical handbook for the health physicist working at a medical institution. As many medical physicists are expected to serve as RSO and provide health physics services to their institutions, the book may also be useful to many in the medical physics community.

The book is divided into chapters on the following topics:

Chapter 1: Radiation Protection in a Medical Institution

Chapter 2: Patient Dosimetry in Diagnostic Imaging

Chapter 3: An Introduction to Diagnostic Radiographic Equipment

Chapter 4: Instrumentation and Radiation Safety Considerations for Nuclear Medicine and Positron Emission Tomography.

Chapter 5: Health Physics in Brachytherapy: Applications of Radiological Safety, Clinical Implementation, and Medical Research

Chapter 6: A Guide for Licensing a Medical Facility for the Use of Radioactive Materials

Chapter 7: The Role of the Health Physicist in Human Biomedical Research

Chapter 8: Medical Events and the Health Physicist

Chapter 9: NRC Initiatives on National Source Tracking

Chapter 10: Radioactive Waste Management in Medical Programs

Chapter 11: Institutional Safety Program for Health Care Laser Systems

Chapter 12: Calculation of Transmission Functions Using NCRP Report No. 147 Methodology

Chapter 13: Linear Accelerator Shielding: Thirty Years Beyond NCRP 49

Chapter 14: Design Elements of a Medical/Radiological Emergency Response Plan

Chapter 15: Radiation Control Agency Inspections at Medical Facilities

Chapter 16: Performing Risk‐Informed, Performance‐Based Radiation Safety Audits at Healthcare Facilities

Chapter 17: Health Effects of Ionizing Radiation Exposure in Medicine: Current Issues

Chapter 18: Running an Effective Hospital Health Physics Program

The book covers a broad range of topics and while some chapters do not add much to information found in literature with which most medical physicists would be familiar, many cover topics to which medical physicists are not generally exposed.

When considering the book as a resource for medical physicists undertaking RSO / health physics responsibilities for the first time, several chapters stand out. Chapter 6 was an excellent and practical guide to Radioactive Materials Licensing. The author gives specific advice on what (and what not) to include in a License application as well as arguments to use to keep from being pushed into adding burdensome License conditions. Chapter 8 gives several examples of Medical Events and lists the facility's corrective actions. This is very useful information for those designing their own programs and trying to anticipate potential sources of errors. Chapters 10 and 11 were thorough treatments of important topics with which many, if not most, medical physicists are not routinely involved. Chapter 15 gives detailed suggestions on preparing for NRC / state inspections and Chapter 18 shares the wisdom gained from the author's personal experiences as RSO at a large facility.

Chapter 13 was a nice summary of the shielding methodology found in McGinley's book and Chapter 2 focused on the patient dosimetry methods described in Wagner et al. but neither chapter added much to the information found in those references.

Chapter 14 gives a good ‘big picture’ overview of how to design an emergency response plan, but more specific examples of how to implement individual procedures would have been appreciated. Specific examples of what did or did not work well during drills would also have been very useful for those trying to put together an emergency response plan.

In summary, Medical Health Physics is a useful resource for medical physicists who are RSOs. The book covers a wide range of topics and provides practical advice and the benefits of experience to those who find themselves undertaking health physics duties at their facility.

